# A novel bidirectional clustering algorithm based on local density

**DOI:** 10.1038/s41598-021-93244-2

**Published:** 2021-07-09

**Authors:** Baicheng Lyu, Wenhua Wu, Zhiqiang Hu

**Affiliations:** 1 State Key Laboratory of Structural Analysis of Industrial Equipment, Dalian University of Technology, Dalian, 116024 China; 2 School of Engineering, Newcastle University, Newcastle Upon Tyne, NE1 7RU UK; 3 Ningbo Institute of Dalian University of Technology, Ningbo, 315000 China

**Keywords:** Information technology, Statistics

## Abstract

With the widely application of cluster analysis, the number of clusters is gradually increasing, as is the difficulty in selecting the judgment indicators of cluster numbers. Also, small clusters are crucial to discovering the extreme characteristics of data samples, but current clustering algorithms focus mainly on analyzing large clusters. In this paper, a bidirectional clustering algorithm based on local density (BCALoD) is proposed. BCALoD establishes the connection between data points based on local density, can automatically determine the number of clusters, is more sensitive to small clusters, and can reduce the adjusted parameters to a minimum. On the basis of the robustness of cluster number to noise, a denoising method suitable for BCALoD is proposed. Different cutoff distance and cutoff density are assigned to each data cluster, which results in improved clustering performance. Clustering ability of BCALoD is verified by randomly generated datasets and city light satellite images.

## Introduction

Cluster analysis is a data processing algorithm using unsupervised learning that has been widely used in machine learning and information recognition^1^. The main clustering methods used so far include K-means clustering, the Gaussian mixture model (GMM), hierarchical clustering, mean shift clustering, and density-based spatial clustering of applications with noise (DBSCAN)^2,3^. K-means clustering can achieve good clustering performance for spherical datasets and is one of the most widely used clustering methods^4^. However, K-means clustering has poor clustering ability in dealing with aspheric data, and it must determine the number of clusters in advance^5,6^. GMM can calculate the expectation and variance of Gaussian datasets^7^, but it is difficult to use for effective cluster analysis of data with poor Gaussian characteristics. Mean shift clustering^8^ can automatically determine the number of clusters, but for a small-cluster dataset the selection of the sliding window radius may affect the clustering results. DBSCAN does not need to determine the number of clusters and can do cluster analysis for data with arbitrary shapes^9^. However, if the density of the sample set is not uniform, and the differences in cluster spacing vary somewhat, the clustering quality is poor and too many parameters must be adjusted. Rodriguez et al. Rodriguez A and Laio A proposed a density-based fast searching method^10^ that overcomes the drawbacks of conventional data clustering methods and can quickly cluster aspheric sets. Unlike the mean shift method, their procedure does not require embedding the data in a vector space; however, it has the problem of requiring manual work to select the number of clusters and easily ignores small clusters, making clustering results depend on manual experience and subjective judgment.

As cluster analysis is applied in more and more fields, the amount of sample data increases, and the cluster number also increases. The increase in the cluster number increases the difficulty in judging the number of clusters for various clustering methods. Small clusters denote points in the data sample that are far away from large clusters and have aggregation phenomenon. Small clusters seldom appear, but in the field of engineering structural safety analysis^11,12^, small clusters usually indicate extreme or unconsidered working conditions. In some cases, accurately identifying small-cluster information is more important than identifying large clusters. Unfortunately, existing methods focus mainly on forming large clusters, and there is little research on small clusters. Also, existing data clustering algorithms for processing noise rely mainly on an artificial threshold. In cluster analysis, using a unified standard denoising indicator increases the discarding of small clusters because the clustering characteristics of each cluster are different. These problems impose challenges on traditional clustering methods.

In this paper, a bidirectional clustering algorithm is proposed that works by combining the advantages of mean shift clustering and clustering by fast search and find of density peaks. The proposed algorithm is divided into an up process and a down process. In the up process, the local density of different data points is calculated to find high-local-density points nearest to data points, and then data chains are formed from data points ranging from low local density to high local density. In the down process, the highest local-density data points are treated as clustering centers, and then the data chains are merged, all data points are traversed, and finally the clustering operation is completed from high-local-density data to low-local-density data. By comparing K-means clustering, GMM, and BCALoD, it was found that the proposed clustering algorithm can quickly calculate the number of clusters and has good recognition performance for small clusters. In the clustering process, the number of parameters requiring adjustment is reduced to a minimum.

The noise problem was also addressed in this study in real-world data measurement^13^ by using the characteristics of a large range and the non-evident aggregation property of noise. A noise reduction decision indicator is proposed that is suitable for BCALoD and assigns a different cutoff distance and cutoff density for each cluster to retain important data as much as possible. By clustering random data examples and city light photographs, it was found that the proposed algorithm has good denoising performance.

## Bidirectional clustering algorithm based on local density (BCALoD)

**Figure 1:**
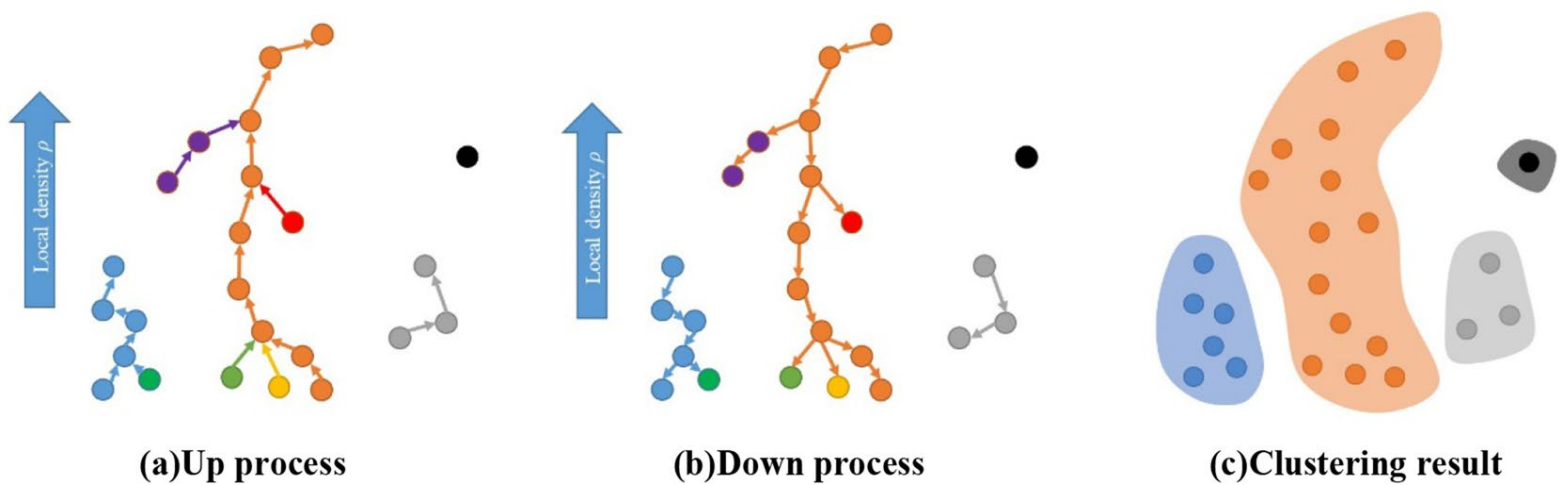
Bidirectional clustering algorithm based on local density. (**a**) Up process. Starting from the data point with minimum local density (*q*_*i*_), the data points *q*_*j*_ whose local density is greater than *ρ*_*i*_ and closest to the *q*_*i*_ point must be searched for. The search operation is repeated until there is no data point with a larger local density within the cutoff distance *d*_*c*_ to form a data chain from *q*_*1*_ to *q*_*m*_. When the same calculation is done for the remaining data, the data chains of all data points are finally obtained. (**b**) Down process. The clustering operation is finally completed by sorting out the data chains formed in the up process, classifying those data chains by their top data points, merging them, and then traversing all the points in the dataset, as shown in (**c**) clustering result.

Assume that the clustering center is data points with maximum local density, and then calculate the local density (*ρ*_*i*_) of each point in the dataset:

1 $$\rho _{i} = \sum\limits_{{j \ne i}} {e^{{ - \left( {\frac{{d_{{ij}} }}{{d_{c} }}} \right)^{2} }} }$$

where *d*_*ij*_ is the Euclidean distance between point *j* and point *i*, and *d*_*c*_ is the cutoff distance selected in the proportion of 1% to 2% of the total distance of data points. Because a Gaussian kernel is used to represent the local distance *ρ*^8,14^, the probability that the local density is the same at all points in the area is low. Assuming that the local density *ρ* of each point is different, data points are sorted according to the value of the local density *ρ*. Figure 1 shows the schematic diagram of BCALoD.

In the up process, the condition for judging point *k* as a cluster center is given by within the cutoff distance, no point has a larger local density than point *k*. For all points in the dataset, each point establishes a connection with only its upper layer, and there is no relation with the next layer; that is, the tops of the selected data chains have nothing to do with the starting point. The up process is started by selecting from the point with the smallest local density *ρ*. The addressing sequence reflects only the path from bottom to top and does not change the local density of the cluster centers. For small clusters, if no point within the cutoff distance has a higher local density, those clusters are formed as one category respectively. Overall, the up process can be regarded as the inverse operation of clustering data points in a density peak clustering algorithm. Algorithm 1 and 2 show the pseudo code of up process and down process, respectively.
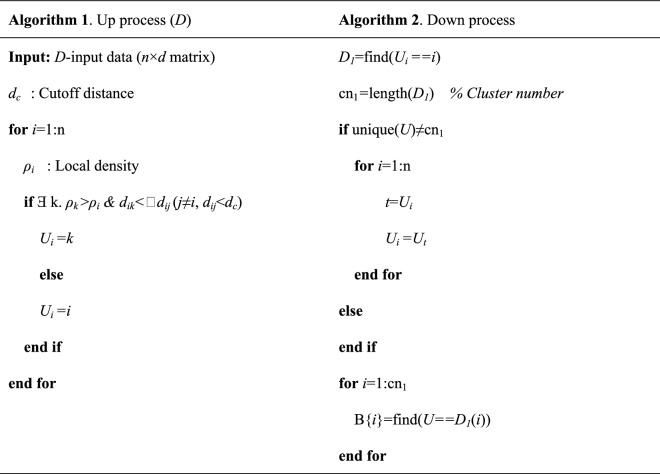


In clustering by fast search and find of density peaks, when the data sizes of large clusters are much greater than those of small clusters, the information of small clusters is easily overwhelmed, resulting in subjectivity in determining the number of clusters. BCALoD can properly identify the clustering of small clusters. The mean shift algorithm must embed random data points and continuously iterate the sliding window, which leads to a high data calculation cost and usually hampers analyzing high-dimensional clusters. The proposed method of constructing data clusters by data chains can reduce the operational cost and ensure that the selection of clustering centers is unrelated to the initial selection. BCALoD establishes the data chains, is not required to embed the data into the vector space, can automatically determine the number of clusters, is more sensitive than other methods to small clusters, and reduces the adjusted parameters to the lowest. In summary, this method combines the advantages of clustering by fast search and find of density peaks and mean shift clustering.

**Figure 2:**
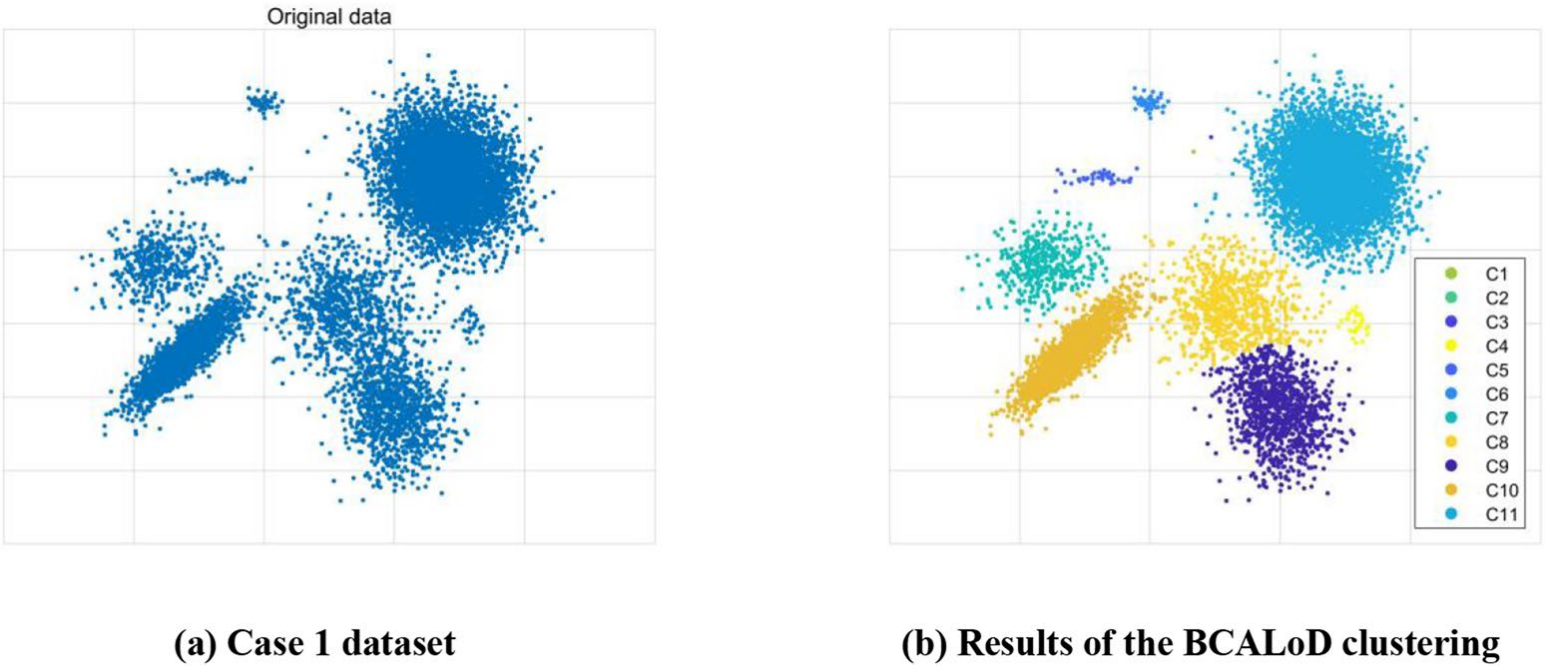
Cluster analysis of 2D Gaussian mixture distribution datasets. (**a**) 2D Gaussian mixture distribution datasets. (**b**) Clustering results of the BCALoD algorithm.

We used a Gaussian mixture distribution to generate a 2D dataset (Case 1), as shown in Fig. 2a. Figure 2b shows the results of the BCALoD clustering algorithm. Table 1 shows the size of each cluster. The largest cluster contains 5,000 data points. While, the size of smallest cluster is only 30. There are 3 small clusters in Case1.

**Table 1:** Cluster sizes of 2D Gaussian mixture distribution datasets.

No	1–3	4	5	6	7	8	9	10	11
Set size	0	30	40	50	400	800	1000	2000	5000
Cluster Result	1	30	41	50	391	747	1036	2016	5006

The resulting clusters 1 through 3 contained one data point each. These data points were distant from the other data points, which are referred to as discrete points. In the BCALoD cluster analysis, such data points are independently grouped into one category. The sizes of cluster 4–6 are 30, 40 and 50, respectively, which are much smaller than the amount of data contained in other clusters; this type of cluster is referred to as a small cluster. The results demonstrate the excellent clustering performance of small clusters by the BCALoD algorithm.

## Noise recognition and cutoff distance optimization

The influence of noise is usually unavoidable in the real world^15^. How to deal with noise reasonably is an important problem in cluster analysis^16^. The noise in cluster analysis has the characteristics of large range and small aggregation. Cluster analysis reveals that empirical values can quickly determine a relatively reasonable range. When the distance between two clusters is artificially increased or noise is added, the values of the cutoff distances also change, whereas the clustering properties in the clusters do not change. Meanwhile, because the aggregation degree of each cluster is different, it is unreasonable to use a uniform cutoff distance for denoising.

**Figure 3:**
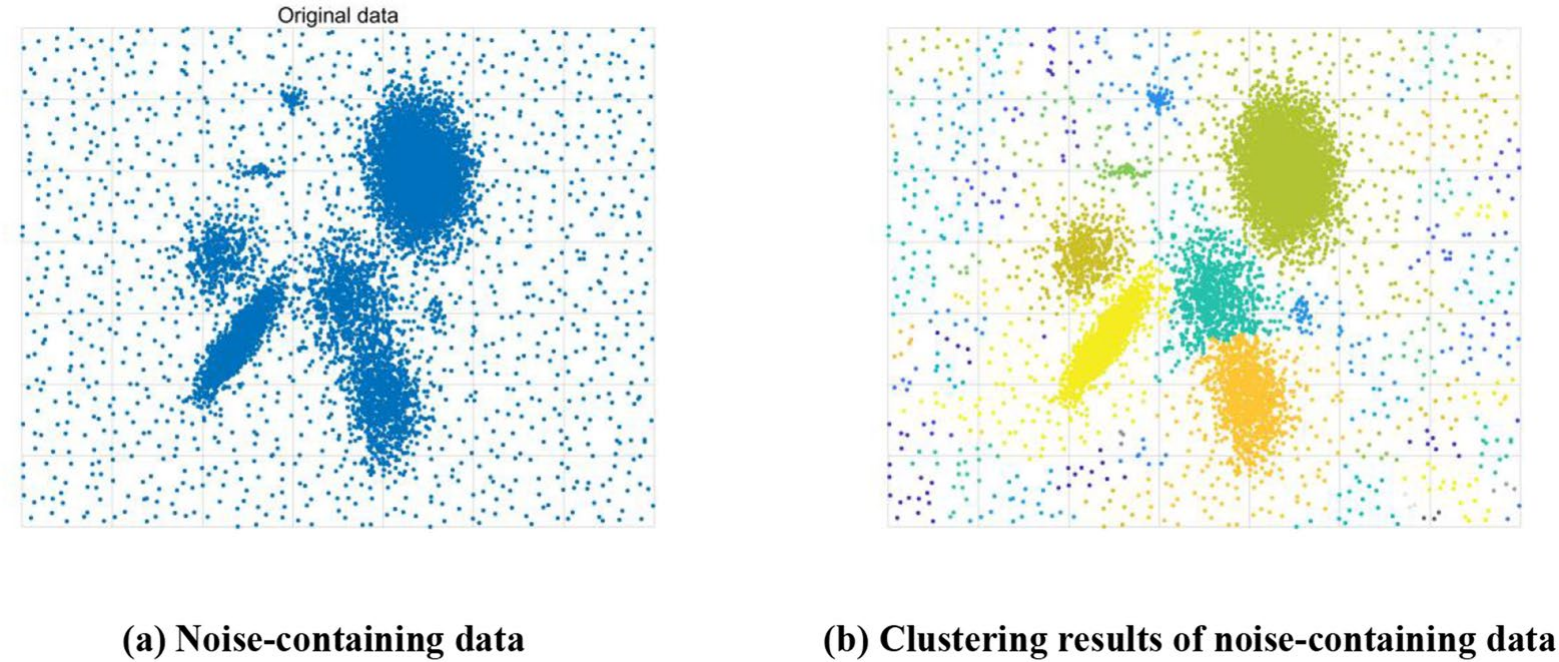
Clustering of a noise-containing 2D Gaussian mixture distribution dataset. (**a**) Noise points were introduced to Case 1. The number of noise points accounted for 11.62% of the whole dataset size. The BCALoD algorithm was used to do cluster analysis for all data, and the *d*_*c*_ value in the case was 1.1833. (**b**) That some noise points were clustered into noise clusters, and the other noise points were clustered into real clusters. The number of clusters was 96.

We introduced noise points to Case 1, as shown in Fig. 3a. The BCALoD algorithm was used to do cluster analysis for all data. Figure 3b shows the clustering results.

**Figure 4:**
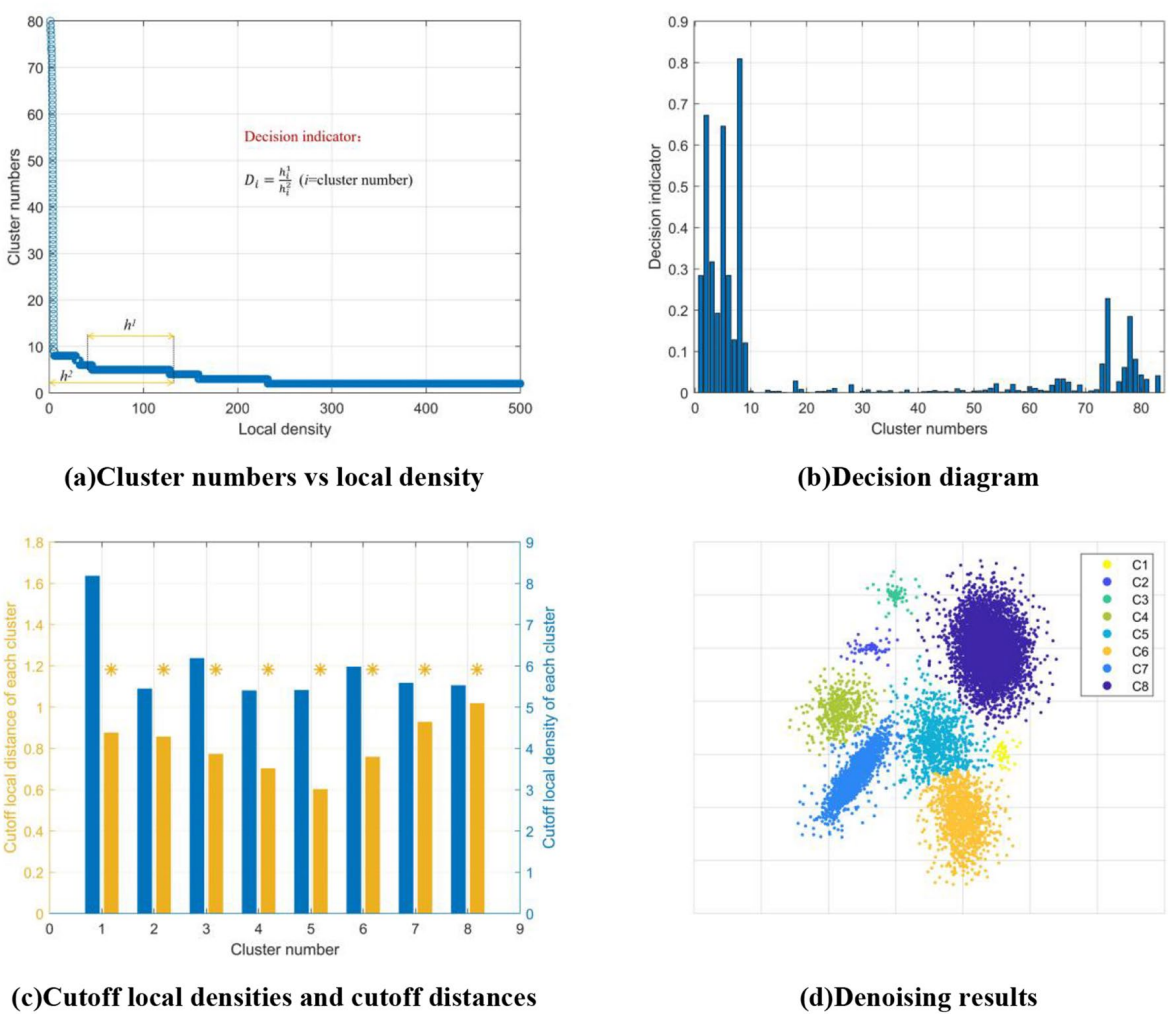
BCALoD noise recognition. (**a**) The number of clusters filtered from small to large local density. When the local density gradually increased, it was observed that the number of clusters gradually decreased. When the local density was small, the number of clusters decreased sharply, and the data points deleted during this period were noise points. When the cutoff local density increased to a certain value, the number of clusters became 1. (**b**) The clustering indicator of the noise-containing 2D Gaussian mixture distribution dataset was calculated. The highest value of the decision indicator *D*_*i*_ was used as the best clustering indicator. To save data points as much as possible, the smallest local density when the decision indicator reached the maximum was selected as the cutoff local density to denoise the dataset. Six clusters were calculated in the dataset. (**c**) Cutoff local densities and cutoff distances of each cluster respectively. There, the yellow point is the initial cutoff distance. (d) The final denoising clustering result calculated by BCALoD. After noise recognition, the number of clusters was corrected form 96 to the true value, 8.

If the number of noise points is much lower than the number of real clusters, denoising can be done simply by comparing cluster sizes (Fig. 2b). However, when there are many real small clusters or noise clusters (Fig. 3b), it is easy to discard real small clusters by denoising using only cluster size. Because of the characteristics of noise, the local density of noise is much lower than that of actual clustering centers. Therefore, when clustering density is gradually filtered from small to large, the low clustering density data points in noise clusters and real clusters are deleted first, while the clustering centers of real clusters are not deleted (Fig. 4a). In other words, the number of clustering centers of real clusters has strong robustness against changes in clustering density.

Set the range of the local density stabilized in *i* clusters as $h_{i}^{1}$. The maximum local density corresponding to the *i* cluster is $h_{i}^{2}$. Set the decision indicator *D*_*i*_ as

2 $$D_{i} = \frac{{h_{i}^{1} }}{{h_{i}^{2} }}$$

If $h_{i}^{1}$ is high, the number of clusters selected as the *i* cluster is stable. If $h_{i}^{2}$ is low, the number of clusters retained is high, which means that more data points are retained. Figure 4b shows the decision indicators of the noise-containing 2D Gaussian mixture distribution dataset. When the cluster number of the dataset is determined, the cutoff local densities and cutoff distances of each cluster can be calculated through data filtering (Fig. 4c), and then the clustering results can be obtained (Fig. 4d). Algorithm 3 presents the process of noise recognition.
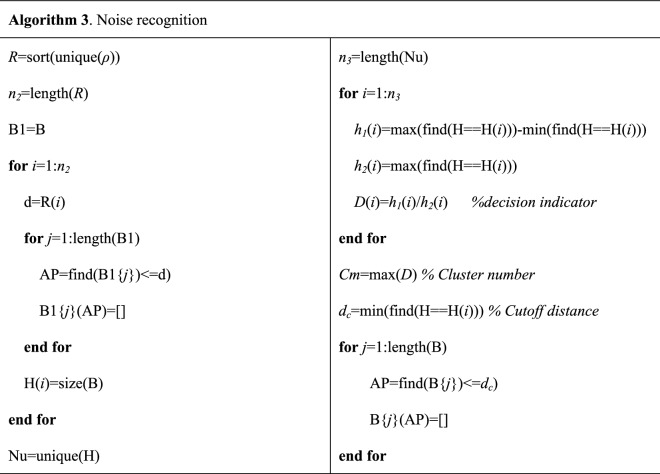


BCALoD algorithm could retain more sensitivity to small clusters and save non-noise data to the greatest extent possible. Also, the algorithm used a decision indicator to scientifically assign a different cutoff local density and cutoff distance for each cluster. Because the denoising process does not require repeated calculations on data points, this algorithm can reduce calculation costs.

## Results and discussion

**Figure 5:**
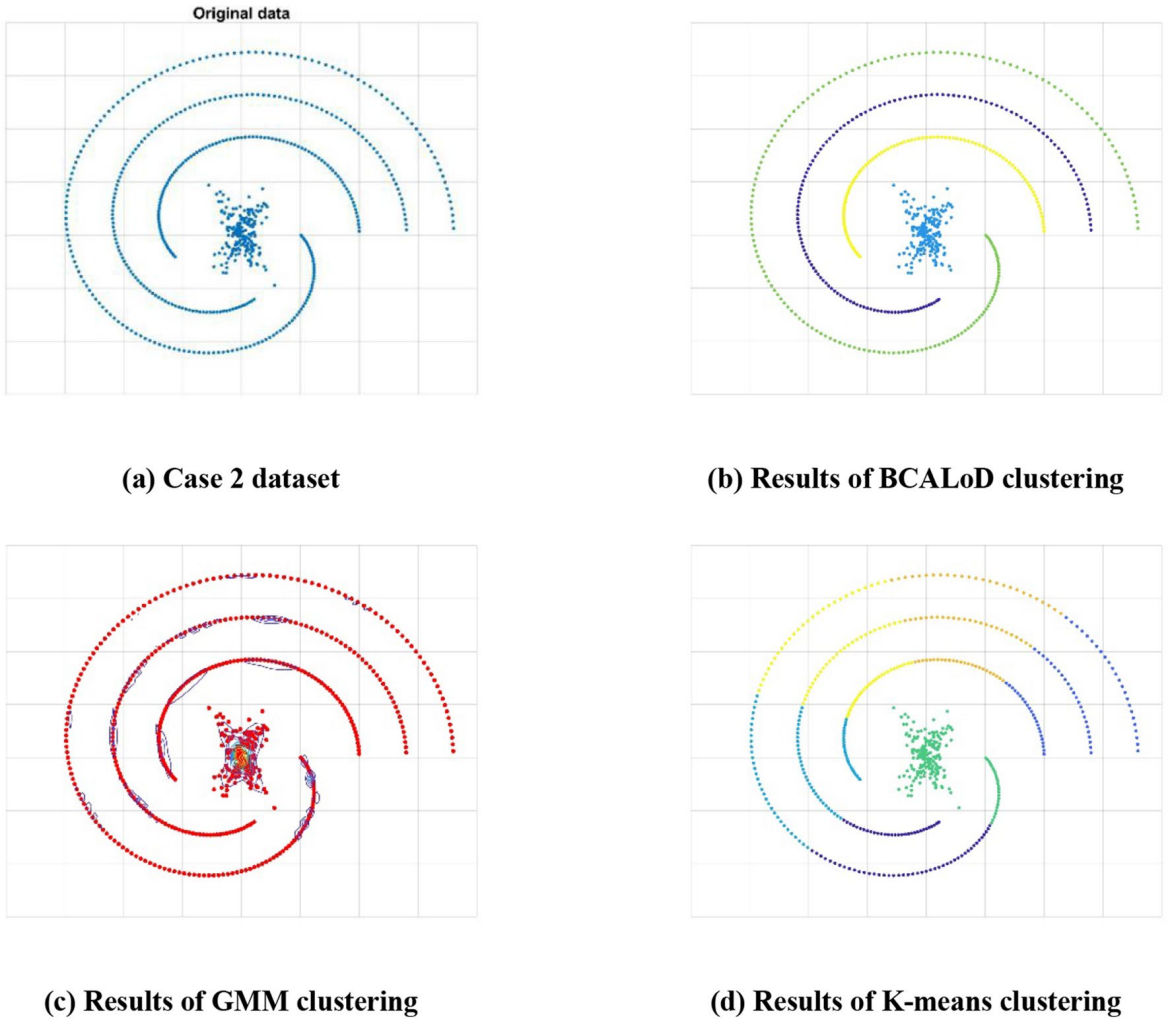
Combination dataset cluster analysis. (**a**) Data points comprised a Gaussian mixture distribution and spirals. (**b**) Accurate numbers of data points in different clusters were obtained by using the BCALoD algorithm. (**c**, **d**) GMM and K-Mean were used to do cluster analysis for the combination dataset. For non-Gaussian spirals GMM could not calculate the expectation and variance of the dataset. Also, the K-mean method has poor clustering result for spirals data points. In this case, neither the silhouette coefficients nor the BIC could accurately provide the optimal solution of the cluster number, and determining the cluster number was difficult. However BCALoD cluster algorithm could effectively cluster the dataset.

We used a combination dataset to verify the BCALoD algorithm by comparing the results of different clustering algorithms, as shown in Fig. 5. By comparing the BCALoD algorithm, K-means clustering, and mixed Gaussian clustering, the relative performance of the BCALoD algorithm was determined. In K-means clustering, the silhouette coefficient^17^ is used to calculate the clustering and separation degrees, and the maximum value of the silhouette coefficient is selected as the number of clusters, which completes K-means clustering. In mixed Gaussian clustering, the Bayesian information criterion (BIC)^18^ is used to select the number of clusters, and the minimum value of the BIC is selected as the data number of clusters.

**Figure 6:**
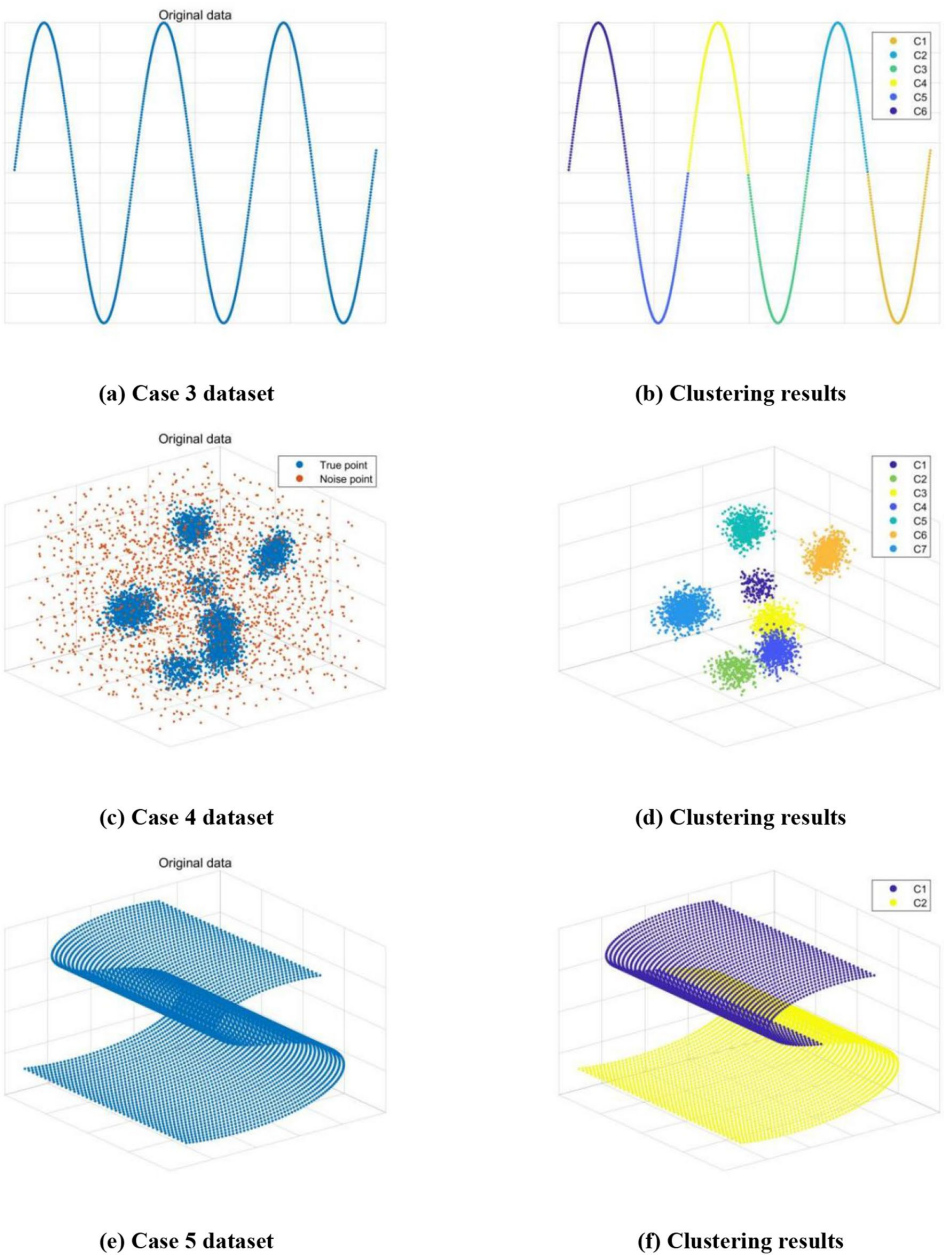
Results of BCALoD clustering.

Table 2 shows the clustering quality indexes (AR, ARI, NMI,HI) of BCALoD, DBSCAN, DPC and K-mean. AR, ARI, NMI and HI are Adjusted Rand Index, Rand Index, Normalized Mutual Information and Hubert index, respectively. When the value of the clustering quality index is close to 1, the clustering effect is closer to the reality. Nc is the number of clusters after calculation. Ns is the set value. All parameters of BCALoD are larger than those of other clustering algorithms, which indicates that BCALoD algorithm has strong clustering ability and efficient noise processing effect on these dataset. Figure 6 shows the clustering results of a variety of 2D and 3D datasets. Algorithm BCALoD presents good clustering ability for different types of datasets.

**Table 2:** Performance of BCALoD, DBcsan, DPC and K-mean. The bolded values are the best clustering quality indexes.

	Algorithm	AR	ARI	NMI	HI	Nc/Ns
Case 1 dataset	BCALoD	**0.9834**	**0.9924**	**0.9481**	**0.9848**	**8/8**
	DBSCAN	0.9367	0.9709	0.8807	0.9418	6/8
	DPC	0.9796	0.9907	0.9350	0.9813	6/8
	K-mean	0.8631	0.9351	0.7918	0.8701	4/8
Case 1 dataset (Noise-containing)	BCALoD	**0.9220**	**0.9674**	**0.8533**	**0.9348**	**8/8**
	DBSCAN	0.8689	0.9444	0.7923	0.8888	7/8
	DPC	0.8536	0.9374	0.7767	0.8748	6/8
	K-mean	0.7639	0.8952	0.6616	0.7904	4/8
Case 2 dataset	BCALoD	**1**	**1**	**1**	**1**	**4/4**
	DBSCAN	1	1	1	1	**4/4**
	DPC	1	1	1	1	**4/4**
	K-mean	0.4293	0.7928	0.3792	0.5855	6/4

For image clustering, pixel values of different data points are different. Because the pixel value of *L*_*i*_ is an integer, it can easily yield the same local density. Therefore, taking the pixel value as the main factor, the local density was defined as:

3 $$P_{i} = \frac{{\rho _{i} }}{{\max (\rho )}} + L_{i}$$

where *ρ*_*i*_ is the local density calculated by Eq. (1). Because *L*_*i*_ is an integer, the value of *ρ*_*i*_ of each point was normalized to make the local density of different data points different. Under the condition of the same pixel, data points were sorted by comparing *ρ*_*i*_.

**Figure 7:**
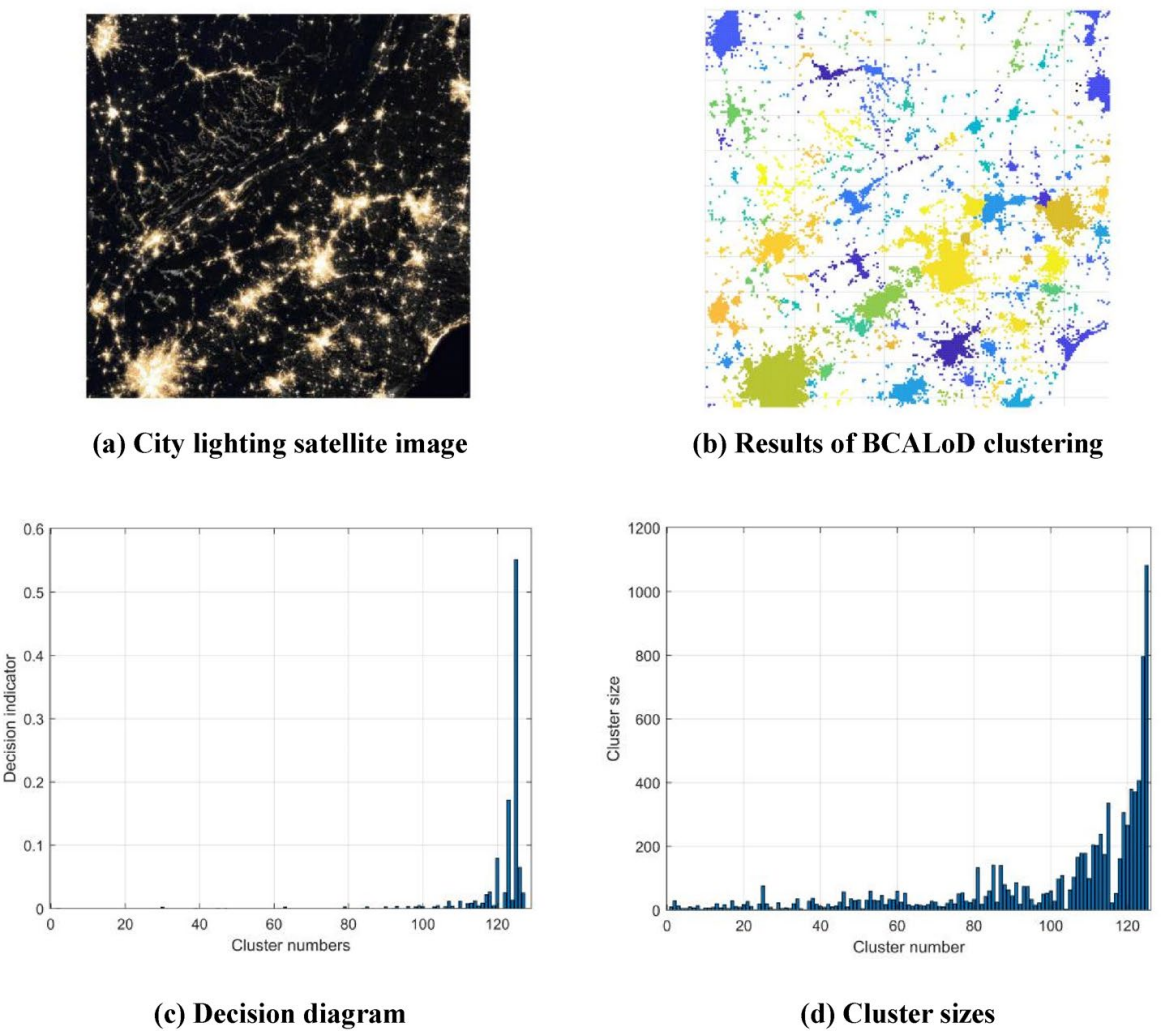
Clustering results of city lighting satellite image. (**a**) A NASA city light satellite image was selected as the dataset. This was also done to verify the BCALoD algorithm’s ability to cluster datasets with multiple cluster centers and small clusters. (**b**) The BCALoD algorithm was used to do cluster analysis on the images, and the clustering results obtained. (**c**) Decision diagram of satellite image BCALoD clustering. The number of clusters before calculate the decision indicators was 128. In the denoising process, a large number of background data points with low local density were removed, and 3 clusters were labeled as noise clusters. Decision indicator reached the maximum value when the number of clusters was 125. (**d**) Cluster size. The size of each cluster was significantly different. The amount of data in the largest cluster (Cluster 125) is 541 times that of the smallest clusters (Cluster 9,36). Using a clustering calculation, the total value of light brightness in each region was obtained. Number of city light clusters reached 125, indicating that the BCALoD algorithm has good clustering capability for datasets of multiple clustering centers. Because the denoising process does not require repeated calculations on data points, BCALod can quickly determine the number of clusters.The results also show that the BCALoD algorithm has accurate clustering performance for data clusters of various shapes, and that small-cluster datasets can be clustered separately using this algorithm.

Figure 7 shows a city lighting satellite image^19^ and the clustering results by BCALoD cluster algorithm. The brightness of each point in the area was different, and the lights were widely distributed. There were many distribution centers in the image, and the distribution shapes were irregular. It would be very difficult to execute effective clustering calculations for these images using traditional methods.

As shown in cases above, for nonspherical clusters, spirals datasets, noise-containing datasets, and city light image data, the proposed algorithm achieved good clustering performance. The only parameter that required adjustment in the clustering calculation was cutoff density, which reduced the difficulty of parameter adjustment in the clustering process as much as possible. The number of clusters was automatically determined by the method based on BCALoD, which was more sensitive to the information of small clusters, and assigned a different cutoff distance and cutoff local density for each data cluster.

Compared with other clustering methods, BCALoD is more sensitive to small clusters and has good denoising ability. Due to the assumption that the clustering center is the data point with the maximum local density, BCALoD clustering algorithm has a relatively weaker effect on the closed annular data clustering than other multi-parameter clustering methods. Therefore, post-processing method of clustering center still needs to be further analyzed. At the same time, BCALoD is relatively sensitive to the local density of the data set, requires a high degree of data density. If the density in the cluster is not high enough, it is easy to judge the data as noise data. The preliminary determination of the cutoff distance *d*_*c*_ still needs further exploration.

## Conclusions

A BCALoD algorithm is proposed in this paper that is based on local density that combines the merits of clustering by fast search and find of density peaks and mean shift clustering. The algorithm forms data chains from low-local-density data points to high-local-density data points, treats the latter as clustering centers, and then integrates the data chains and completes the clustering operations. The number of clusters is automatically determined by the BCALoD algorithm, which is more sensitive to small clusters, and reduced the number of parameters requiring adjustment to the lowest.

Using the characteristics of noise, a denoising method is proposed based on local density, which ensures a denoising effect and retains the sensitivity of the BCALoD algorithm to small clusters. The denoising method assigns a cutoff local density and cutoff distance for each cluster, retaining useful data as much as possible. The proposed algorithm can also reduce calculation cost.

The BCALoD algorithm can do effective cluster analyses on aspheric clusters, spirals datasets, noise-containing datasets, and image data. The proposed algorithm can also achieve ideal clustering performance for clusters of various shapes. When the number of clusters is large, the BCALoD algorithm can quickly determine the number of clusters, is more sensitive to small clusters, and removes noise effectively.
